# Effect of Cytomegalovirus (CMV) and Ageing on T-Bet and Eomes Expression on T-Cell Subsets

**DOI:** 10.3390/ijms18071391

**Published:** 2017-06-29

**Authors:** Fakhri Hassouneh, Nelson Lopez-Sejas, Carmen Campos, Beatriz Sanchez-Correa, Raquel Tarazona, Alejandra Pera, Rafael Solana

**Affiliations:** 1 Immunology Unit, IMIBIC-Hospital Universitario Reina Sofia-Universidad de Cordoba, Córdoba 14004, Spain; hassounehfakhri@yahoo.com (F.H.); nelsonj836@hotmail.com (N.L.-S.); mccampos1977@gmail.com (C.C.); 2 Immunology Unit, Department of Physiology, University of Extremadura, Cáceres 10003, Spain; beatrizsanchezcorrea@gmail.com (B.S.-C.); rtarazon@unex.es (R.T.); 3 Division of Clinical and Experimental Medicine, Brighton and Sussex Medical School, Brighton BN1 9PS, UK

**Keywords:** ageing, cytomegalovirus (CMV), CD57, Eomes, T-bet, T-cells

## Abstract

The differential impact of ageing and cytomegalovirus (CMV) latent infection on human T-cell subsets remains to some extent controversial. The purpose of this study was to analyse the expression of the transcription factors T-bet and Eomes and CD57 on CD4+, CD4^hi^CD8^lo^ and CD8+ T-cell subsets in healthy individuals, stratified by age and CMV serostatus. The percentage of CD4+ T-cells expressing T-bet or Eomes was very low, in particular in CD4+ T-cells from young CMV-seronegative individuals, and were higher in CMV-seropositive older individuals, in both CD57− and CD57+ CD4+ T-cells. The study of the minor peripheral blood double-positive CD4^hi^CD8^lo^ T-cells showed that the percentage of these T-cells expressing both Eomes and T-bet was higher compared to CD4+ T-cells. The percentage of CD4^hi^CD8^lo^ T-cells expressing T-bet was also associated with CMV seropositivity and the coexpression of Eomes, T-bet and CD57 on CD4^hi^CD8^lo^ T-cells was only observed in CMV-seropositive donors, supporting the hypothesis that these cells are mature effector memory cells. The percentage of T-cells expressing Eomes and T-bet was higher in CD8+ T-cells than in CD4+ T-cells. The percentages of CD8+ T-cells expressing Eomes and T-bet increased with age in CMV-seronegative and -seropositive individuals and the percentages of CD57− CD8+ and CD57+ CD8+ T-cells coexpressing both transcription factors were similar in the different groups studied. These results support that CMV chronic infection and/or ageing are associated to the expansion of highly differentiated CD4+, CD4^hi^CD8^lo^ and CD8+ T-cells that differentially express T-bet and Eomes suggesting that the expression of these transcription factors is essential for the generation and development of an effector-memory and effector T lymphocytes involved in conferring protection against chronic CMV infection.

## 1. Introduction

The ageing of the immune system, referred as immunosenescence, has been observed in both multicellular invertebrate and vertebrate organisms, including humans [1]. As chronological age advances, the immune system becomes less efficient at reacting against pathogens. It has been described that situations involving chronic activation of the immune system, such as viral infections or cancer, can induce changes in the immune system, referred as “early immunosenescence” [2,3,4,5]. Among viral infections, persistent cytomegalovirus (CMV) infection has been described to be involved in the process of immunosenescence, contributing to the detriment of the immune response observed in elderly donors. Thus, it is well known that CMV infection impacts on the T-cell compartment in ageing [6,7,8,9].

The transcription factors T-bet (T-box expressed in T-cells) and Eomesodermin (Eomes) belong to the T-box family of transcription factors that play crucial roles in the development of different organs and tissues including the immune system. The studies of T-bet and Eomes in the context of human T-cells are relatively limited. In murine models T-bet was originally involved in promoting Th1 CD4+ T-cell development while inhibiting the Th2 [10] and Th17 lineage commitment [11,12]. T-bet is also known to modulate a number of genes involved in T-cell trafficking and function [13]. Thus, high levels of T-bet have been associated with superior cytotoxic capacity of CD8+ T-cells by upregulation of perforin and granzyme B expression [14,15]. Although T-bet has been involved in sustaining memory T-cells [14], it has been reported that low expression of T-bet shifts the cells into memory-like cells impaired in sensing homeostatic signals from IL-15 [16,17].

The role of T-bet and Eomes in immune cell development and cytolytic function has been previously described. These transcription factors are expressed in different human blood cell subsets, including CD4+ and CD8+ T-cells, γδ T-cells, invariant natural killer (NK) T-cells, NK cells, B cells, and dendritic cells. T-bet expression increases as peripheral cells become more differentiated [18]. Although T-bet and Eomes expression shows some overlap, their functions are not totally reciprocal. In human peripheral blood T-cells, T-bet expression increases as cells differentiate from naive to effector-memory and effector cells. In contrast to CD8+ T-cells, the expression of T-bet and Eomes is very low in CD4+ T-cells. In addition, whereas the percentages of naive cells CD4 and CD8 T-cells expressing T-bet or Eomes is very low, a high percentage of effector-memory and effector CD4 and CD8 T-cells express these factors and the analysis of T-bet and Eomes coexpression shows that in both CD8+ and CD4+ T-cells the majority of effector-memory and effector cells expressing high levels of T bet (T-bet^hi^) coexpress Eomes+ [18]. The relevance of these transcription factors in the differentiation, development and maintenance of antiviral CD8+ T-cell responses in mice and human is well established [19,20,21].

In this study, we sought to further characterize the expression of T-bet and Eomes on human CD4 and CD8 T-cell subsets from healthy human donors in the context of ageing and CMV infection. This analysis included the possible relationship with the expression of CD57, originally defined as a marker of senescent T-cells and more recently considered a marker of highly differentiated effector-memory or effector T-cells responsible of cytokine production.

## 2. Results

### 2.1. Molecular Signature of T-Cells in the Context of Ageing and CMV Infection

**Figure 1:**
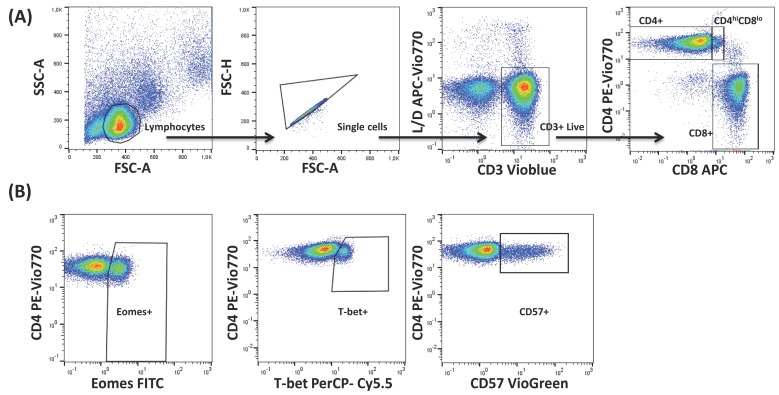
Gating strategy used for the analysis of T-bet, Eomes and CD57 expression on T-cells. (**A**) Peripheral blood lymphocytes (PBLs) were selected using forward (FSC) and side scatter (SSC) detectors. Subsequently Live CD3+ T-cells were gated from PBLs after single cells gating, followed by identification of CD4+, CD8+ and CD4^hi^CD8^lo^ T-cells. Arrows show the sequence of the gating used, starting from the lymphocytes gate. (**B**) Total expression of Eomes, T-bet and CD57 was determined within each T-cell subset using Fluorescence minus one (FMO) controls. Figure shows a representative example for CD4+ T-cells.

Multicolour flow cytometry was used to analyse the percentage of CD4+, CD4^hi^CD8^lo^, and CD8+ T-cells expressing T-bet and Eomes transcription factors, as well as in regard to CD57 expression in those subsets (Figure 1). The analysis was carried out on cells from healthy individuals stratified by age and CMV serostatus (Table 1). FlowJo’s Boolean analysis was used to analyse the coexpression of T-bet and Eomes in combination with CD57. However, not all the possible Boolean combinations contained a significant number of cells.

### 2.2. T-Bet and Eomes Expression in CD4+ T-Cells

**Figure 2:**
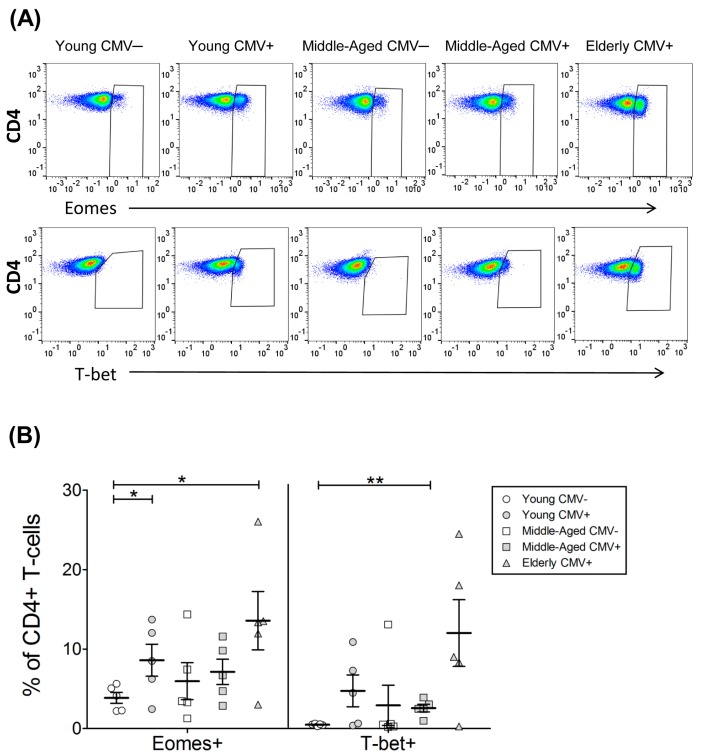
T-bet and Eomes expression on CD4+ T-cells. (**A**) Dot plot graphs representative of each group showing the expression of the studied markers on CD4+ T-cells; (**B**) percentage of CD4+ T-cells expressing T-bet and Eomes on healthy individuals (*n* = 25), stratified by age and CMV serostatus. Scatter plot shows the mean and SEM. Results were considered significant at * p < 0.05 and ** *p* < 0.01.

As some CD4+ T-cells express low levels of CD8 marker and it has been shown to have a mature phenotype distinct from CD4+CD8− T-cells, we decided to analyse both subpopulations separately. Our data showed that although the majority of CD4+CD8− (CD4+) T-cells do not express these transcription factors, in CMV-seropositive and aged individuals CD4+ T-cells expressing both T-bet and Eomes are detectable. Thus, the percentage of cells expressing Eomes increased with CMV infection in young individuals and was higher in the elderly CMV-seropositive (Figure 2, Table 2 and Table S1). The percentage of CD4+T-bet+ T-cells on young CMV-seronegative individuals was null. CD4+ T-cells expressing this transcription factor were higher in CMV-seropositive individuals and older individuals, being particularly high in the older CMV-seropositive individuals.

**Figure 3:**
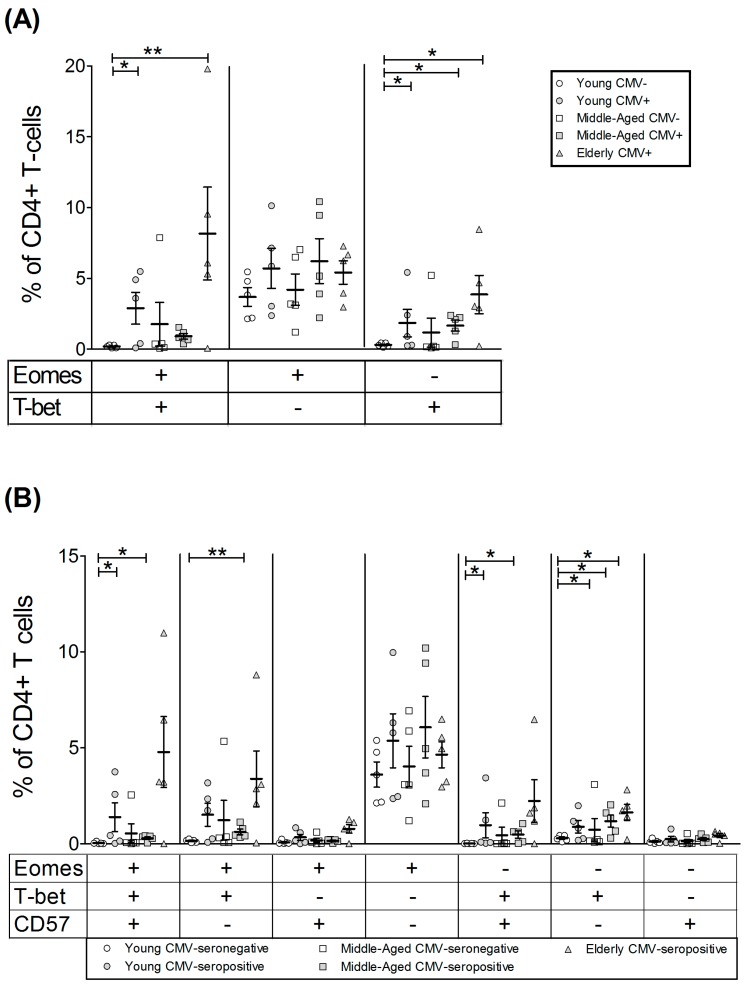
T-bet and Eomes coexpression with and without CD57 on CD4+ T-cells from healthy individuals (*n* = 25), stratified by age and CMV serostatus. (**A**) Coexpression of T-bet and Eomes on CD4+ T-cells; (**B**) T-bet, Eomes and CD57 coexpression on CD4+ T-cells. Scatter plots show the mean and SEM. The combination of markers studied is indicated in the table below the scatter graphs. Results were considered significant at * p < 0.05 and ** *p* < 0.01.

The Boolean combination of both transcription factors in CD4+ T-cells showed that the percentage of cells expressing Eomes but not T-bet was not significantly affected by age or CMV infection (Figure 3A). Cells expressing only T-bet were less frequent and increased in CMV-seropositive individuals and further increased with age. In a similar way, double-positive Eomes+ T-bet+ cells were very low in young CMV-seronegative individuals and increased in CMV-seropositive donors with maximum values in the elderly CMV-seropositive.

The analysis of T-bet and Eomes according to CD57 expression on CD4+ T-cells (Figure 3B, Table S2) showed that cells expressing both transcription factors and CD57+ are null in young CMV-seronegative. Moreover, although very variable, the highest percentages of these cells were found in the elderly CMV-seropositive. Indeed, CD57+CD4+ T-cells were only found in combination with T-bet with or without Eomes. The majority of CD57−CD4+ T-cells were Eomes+T-bet−. The percentages of these cells remained stable despite CMV infection and age. The percentage of Eomes+CD57−CD4+ T-cells was only affected by age when coexpressing T-bet.

### 2.3. T-Bet and Eomes Expression on CD4^hi^CD8^lo^ T-Cells

**Figure 4:**
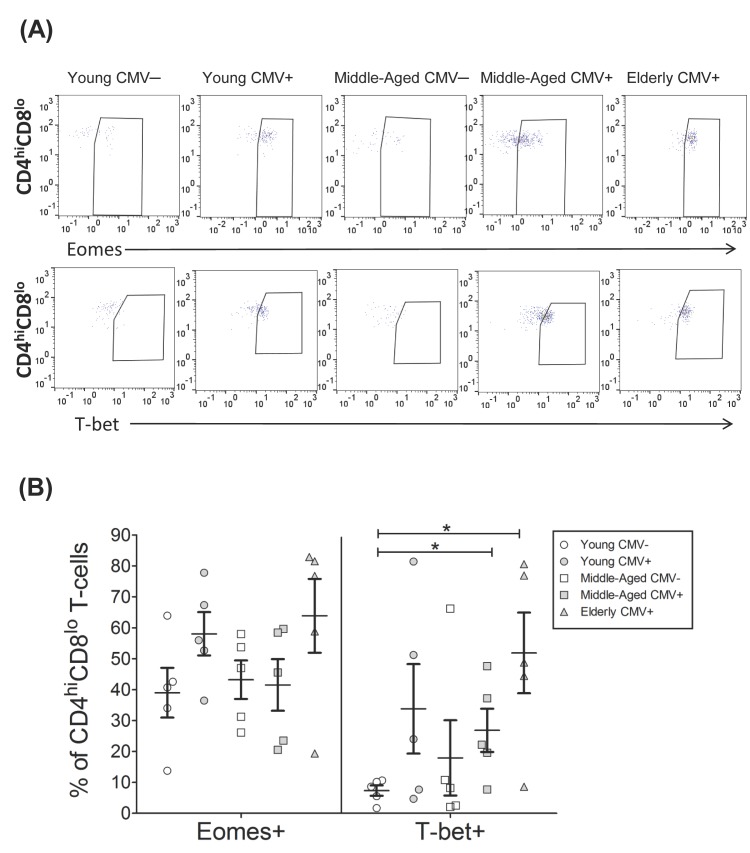
T-bet and Eomes expression on CD4^hi^CD8^lo^ T-cells. (**A**) Dot plot graphs show the expression of T-bet and Eomes on CD4^hi^CD8^lo^ T-cells for each group studied; (**B**) expression of T-bet and Eomes on CD4^hi^CD8^lo^ T-cells from healthy individuals (*n* = 25), stratified by age and CMV serostatus. Scatter plot shows the mean and SEM. Results were considered significant at * *p* < 0.05 and ** *p* < 0.01.

The CD4^hi^CD8^lo^ subset was very low in young CMV-seronegative individuals, and, although there is no significant difference in percentage of cells, the individuals with the highest numbers of these cells were all CMV-seropositive, particularly the elderly (Figure S1). The percentage of CD4^hi^CD8^lo^ T-cells expressing Eomes was over 40% in all groups studied and noticeably higher than those of CD4+ T-cells (below 20%). Eomes+ CD4^hi^CD8^lo^ T-cells percentage was similar in all groups studied. The percentage of T-bet+ CD4^hi^CD8^lo^ T-cells was also higher than in CD4+ T-cells and particularly high in old CMV-seropositive individuals (Figure 4, Table 3).

**Figure 5:**
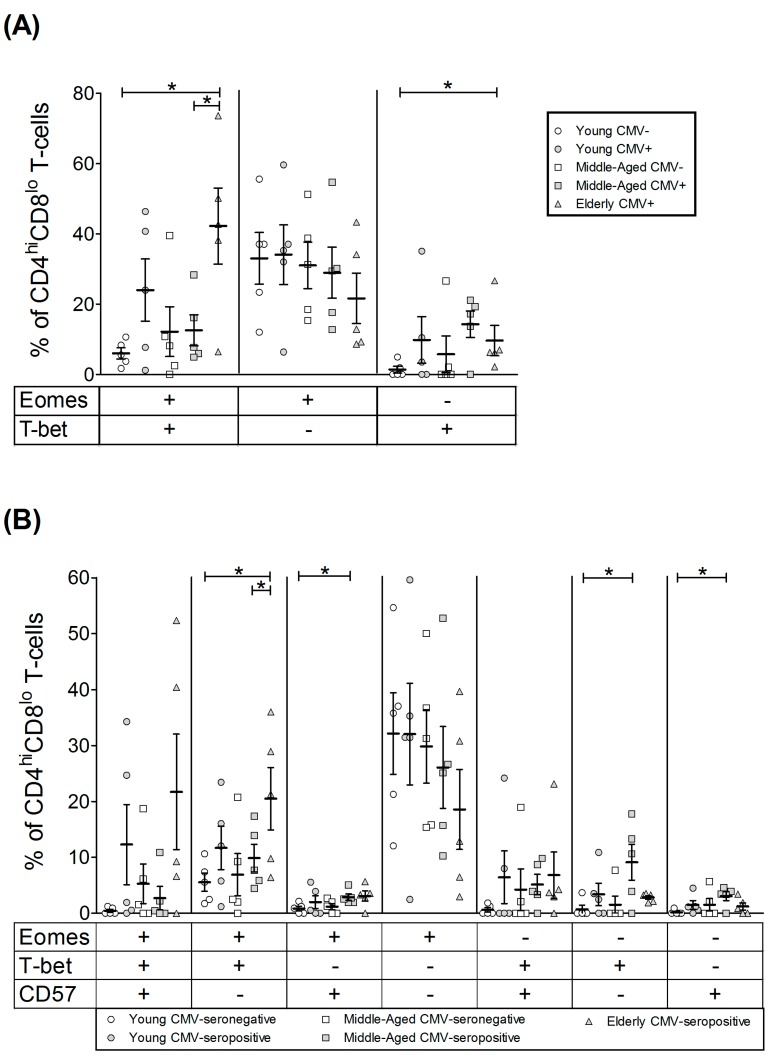
T-bet and Eomes coexpression with and without CD57 on CD4^hi^CD8^lo^ T-cells from healthy individuals (*n* = 25), stratified by age and CMV serostatus. (**A**) Coexpression of T-bet and Eomes on CD4^hi^CD8^lo^ T-cells; (**B**) T-bet, Eomes and CD57 Boolean combinations on CD4^hi^CD8^lo^ T-cells. Scatter plots show the mean and SEM. The combination of markers studied is indicated in the table below the scatter graphs. Results were considered significant at * *p* < 0.05 and ** *p* < 0.01.

Analysis of the coexpression pattern of Eomes and T-bet in CD4^hi^CD8^lo^ T-cell subset showed that the majority of cells from young CMV-seronegative individuals were Eomes+ T-bet− and the percentage of these cells were not affected by CMV infection or age. Cells expressing T-bet were present in significant numbers in the elderly CMV-seropositive individuals, especially the Eomes+ T-bet+ cells (Figure 5A).

The analysis of T-bet and Eomes expression in regards to CD57 expression on CD4^hi^CD8^lo^ T-cells (Figure 5B, Table S2) showed that the percentage of cells expressing both transcription factors and CD57 was very low in the young CMV-seronegative individuals and values were significantly higher in elderly seropositive donors. CD57+ CD4^hi^CD8^lo^ T-cells always coexpressed T-bet with or without Eomes. The majority of CD57− CD4^hi^CD8^lo^ T-cells were Eomes+ and were not affected by CMV infection. CD57−Eomes+T-bet+ cells increased with age in CMV-seropositive individuals.

### 2.4. T-Bet and Eomes Expression in CD8+ T-Cells

**Figure 6:**
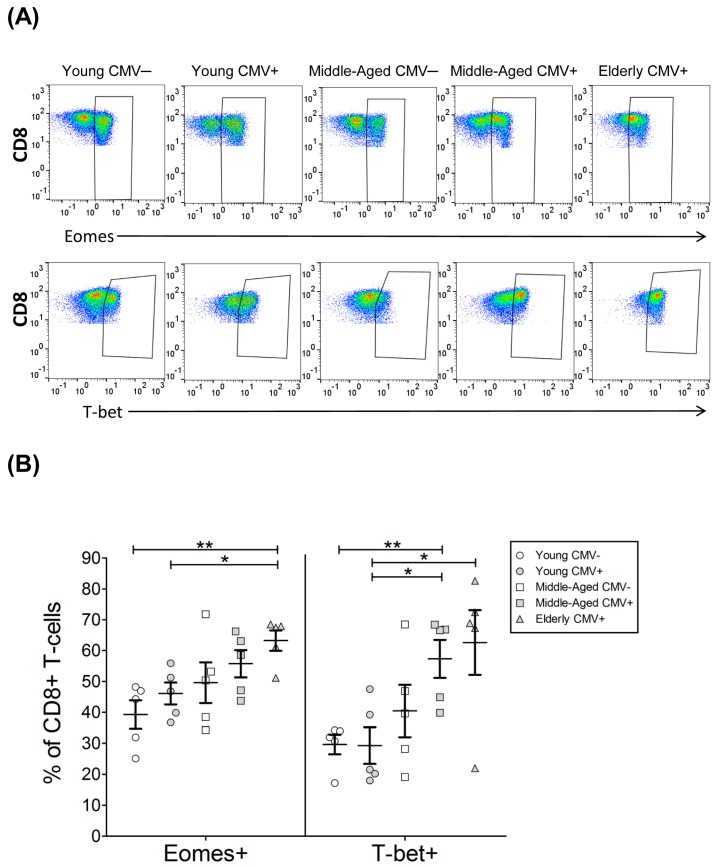
T-bet and Eomes expression on CD8+ T-cells. (**A**) Dot plot graphs representative of each group showing the expression of T-bet and Eomes on CD8+ T-cells; (**B**) T-bet and Eomes expression on CD8+ T-cells from healthy individuals (*n* = 25), stratified by age and CMV serostatus. Scatter plot shows the mean and SEM. Results were considered significant at * *p* < 0.05 and ** *p* < 0.01.

**Figure 7:**
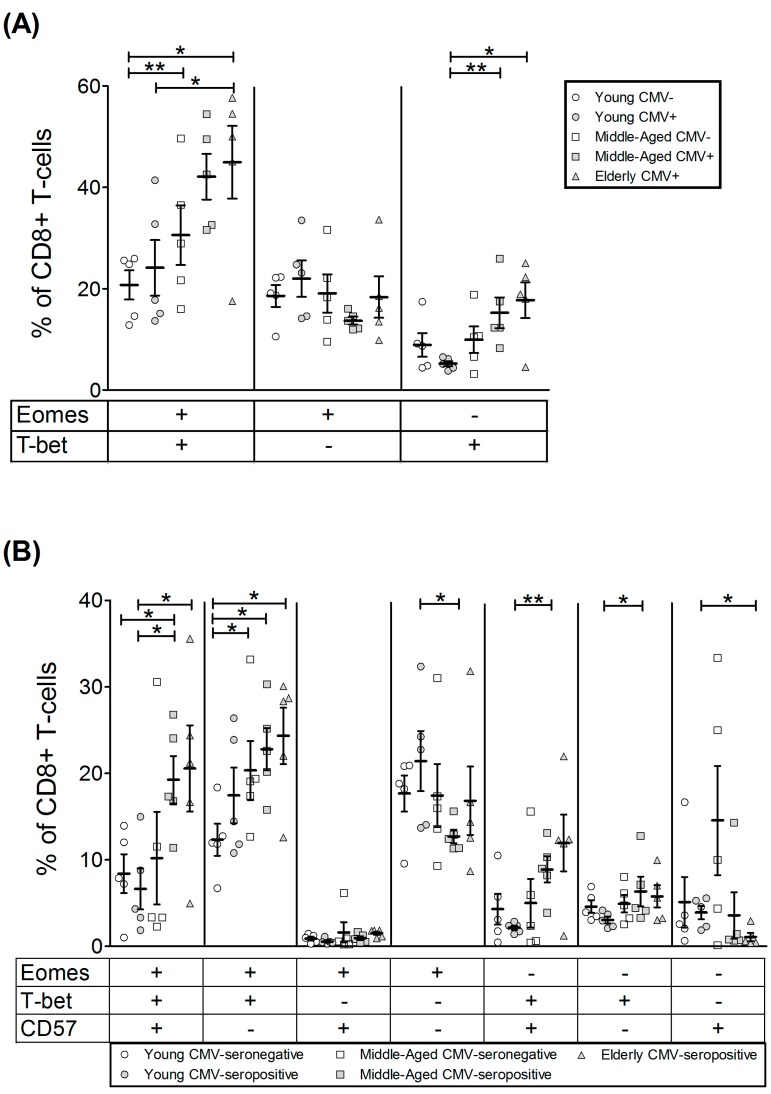
T-bet and Eomes coexpression with and without CD57 on CD8+ T-cells from healthy individuals (*n* = 25), stratified by age and CMV serostatus. (**A**) Coexpression of T-bet and Eomes on CD8+ T-cells; (**B**) T-bet, Eomes and CD57 coexpression on CD8+ T-cells. Scatter plots show the mean and SEM. The combination of markers studied is indicated in the table below the scatter graphs. Results were considered significant at * *p* < 0.05 and ** *p* < 0.01.

As expected, the percentage of CD8+ T-cells expressing T-bet and Eomes was higher than in CD4+ T-cells (Table 3 and Table 4). The fraction of CD8+ T-cells expressing whichever transcription factor increased with age in CMV-seropositive individuals (Figure 6 and Figure S1, Table 4) Furthermore, the percentage of CD8+ T-cells that coexpressed both Eomes and T-bet was higher with age and reaching the highest values in the elderly CMV-seropositive individuals (Figure 7A).

The analysis according to CD57 expression (Figure 7B, Table S2) showed that the percentage of CD8+ T-cells coexpressing Eomes and T-bet increase with age, independently of CD57 expression. CD57+CD8+ T-cells expressed very low levels of Eomes in the absence of T-bet, although a proportion of CD57+CD8+ T-cells expressed T-bet without expressing Eomes. CD57+ CD8+ T-cells that did not express any of the transcription factors studied were very low or null in the elderly.

In summary, CD4+ T-cells expressing CD57 always coexpress T-bet and are only found in CMV-seropositive individuals independently of their age. In young CMV-seronegative individuals we did not observed expression of CD57 or T-bet by CD4+ T-cells. The expansion of these cells is clearly a hallmark of CMV infection and is further increased by age. Besides, CD4^hi^CD8^lo^ T-cells have a different phenotype than CD4+ T-cells, which do not express CD8. This subset contains higher numbers of cells expressing CD57, T-bet and Eomes. These results support the hypothesis that CD4^hi^CD8^lo^ cells derive from CD4+ T-cells and are mature effector memory cells. On the other hand, the percentages of T-bet+CD8+ and Eomes+CD8+ T-cells were similar, increased with age and where higher than those of CD4+ T-cells. The majority of CD8+ T-cells coexpressed both transcription factors with or without CD57. Finally, in all subsets studied CD57+ T-cells were T-bet+ with or without Eomes but never Eomes+T-bet−.

## 3. Discussion

Advances in the last decade on the processes involved in immunosenescence support the idea that some of the accepted hallmarks of T-cell immunosenescence in humans, such as the decreased numbers of peripheral naïve T-cells and the accumulations of memory T-cells (especially affecting the CD8+ T-cell compartment), are determined not only by the lower output of T-cells due to thymus involution but also by the individual’s history of pathogen exposure, particularly to infection with CMV. Thus, it is generally accepted that CMV infection is a major driving force contributing to the age-associated deterioration of adaptive immunity [22,23,24]. One of these hallmarks of human immunosenescence associated with long-term CMV infection is the oligoclonal expansion of late-stage differentiated effector memory or effector T-cells, characterised by the downregulation of co-stimulatory molecules such as CD27 or CD28 and the expression of markers as KLRG-1 and CD57 [25].

CMV seropositivity increases with age in all the populations studied and is influenced by geographic, ethnic and socioeconomic factors [26] and CMV seropositivity has been associated with a significant increased risk of all-cause mortality [27].

Several studies have contributed to defining the expression of the transcription factors T-bet and/or Eomes in T-cells and their significance in controlling CD8+ and CD4+ T-cell functions in mice and human. In particular these transcription factors are involved in the development and maintenance of immune response to different pathogens [21,28].

In this study we characterise the expression of the transcription factors T-bet and Eomes in resting peripheral blood T-cell subsets defined by the expression of CD4, CD8 and CD57, from healthy individuals stratified according to age and CMV serostatus. Several studies have shown that T-bet and Eomes are expressed in human peripheral blood T-cells, with a higher expression on CD8+ than in CD4+ T-cells. In addition, whereas the percentages of naive cells CD4 and CD8 T-cells expressing T-bet or Eomes is very low, a high percentage of effector-memory and effector CD4 and CD8 T-cells express these factors [18].

It has been shown that human peripheral blood CD4+ T-cells are predominantly T-bet^lo^Eomes−, suggesting that in peripheral blood CD4+ T-cells T-bet and Eomes likely do not significantly cooperate to modulate CD4+ T-cell function [18]. The levels of T-bet and Eomes are very low in CD4 naïve CD4 T-cells, and increase as T-cells become more differentiated and those CD4 T-cells that coexpress T-bet and Eomes show an effector or effector-memory phenotype [18]. Our results show that the percentage of resting CD4+ T-cells expressing T-bet T-cell was undetectable in young CMV-seronegative individuals whereas variable percentages of T-bet positive CD4+ T-cells were found in CMV-seropositive young donors, with higher values in the CMV-seropositive elderly individuals, supporting the relevant role of CMV in the generation of effector-memory and effector CD4+ T-cells. The expression of Eomes is also higher in CMV-seropositive (young and old) donors compared with CMV-seronegative young donors. Subsequently, the Boolean analysis of both transcription factors showed that the percentage of CD4+ T-cells coexpressing Eomes and T-bet cells was very low in young CMV-seronegative individuals and increased with CMV seropositivity in young and aged donors. The percentage of CD4+ T-cells expressing T-bet but not Eomes was also negligible in young CMV-seronegative individuals and increased in CMV-seropositive young, middle-aged and elderly donors. No significant changes were found in the percentages of CD4+ T-cells expressing Eomes but not T-bet among the groups studied.

The expression of CD57 on T-cells increases with age [29], and we have previously shown that whereas it is virtually absent on CD4 T-cells from CMV-seronegative young individuals, its expression is higher in CMV-seropositive donors [30]. Although CD57 was originally defined as a marker of senescent CD4 and CD8 T-cells, it is considered a marker of a subset of highly differentiated effector-memory and effector cells. Furthermore, in CMV-seropositive young individuals, CD57+CD4+ T-cells are polyfunctional cells and produce several cytokines, including IFNγ [30]. Since both T-bet and Eomes are known to induce IFNγ expression and other effector functions in CD4 T-cells, we next examined the coexpression of these factors according to the expression of CD57. As expected, no CD4 T-cells coexpressing CD57 and T-bet are found in young CMV-seronegative individuals. The majority of CD57+CD4+ T-cells that are found in the rest of groups studied express T-bet alone or in combination with Eomes, and CD57 expression is negligible in T-bet negative CD4 T-cells. It has been shown that stimulation of CD4 T-cells via TCR and CD28 results in the upregulation of T-bet in most CD4+ T-cells, whereas Eomes is only upregulated on a subset of polyfunctional CD4+ T-cells characterised by the expression of CD300a [31]. Since we have recently shown that CD57 is also a marker of polyfunctional CD4+ T-cells in CMV-seropositive young donors [30] and that the expression of CD300a on CD4+ T-cells is higher in CMV-seropositive aged donors [32], these results support the relevance of CMV infection on the expansion of effector memory and effector polyfunctional CD4+ T-cells that coexpress T-bet and Eomes. In a similar way, it has been shown that CD57+CD4+ T-cells that produce TNF-α and IFN-γ in response to CMV stimulation express higher levels of T-bet and Eomes when compared with CD57−CD4+ T-cells that produce TNF-α and express lower levels of both T-bet and Eomes [33]. The recent demonstration that T-bet induction on CD4+ T-cells in response to CMV stimulation, which correlated with proliferation and effector multifunction, allows us to differentiate lung transplant recipients mismatched for CMV that control CMV replication from those with early relapse [34] supports the relevance of this transcription factor in the generation and maintenance of CD4+ effector cells able to establish effective immune control during the early stages of CMV infection.

Peripheral blood double-positive (DP) CD4+ and CD8+ T-cells constitute a minor subset of T-cells that can be found in healthy individuals of different ages, and their percentage is increased in inflammatory autoimmune disease. An age-dependent increase in the different DP T-cell subsets described has been observed [35]. In particular, CD4^hi^CD8^lo^ (CD8αα) cells have been shown to be terminally differentiated effector CD4+ T-cells that acquire the alpha-chain of CD8 (for revision see [36]). Analysis of peripheral blood CD4^hi^CD8^lo^ T-cells has shown that these cells have a highly differentiated effector-memory phenotype, expressing more CXCR3 and CD57 than CD4+CD8− T-cells [37]. Our results show that the percentage of cells expressing both Eomes and T-bet was higher in the CD4^hi^CD8^lo^ subset than in CD4+CD8− T-cells and that the percentage of CD4^hi^CD8^lo^ T-cells expressing T-bet was associated with CMV-seropositivity. Similar to CD4+ T-cells, the coexpression of Eomes, T-bet and CD57 on CD4^hi^CD8^lo^ T-cells was mainly observed in CMV-seropositive donors. The analysis of T-bet and Eomes on CD4^hi^CD8^lo^ T-cells supports the notion that these cells represent a subset of highly differentiated CD4+ T-cells that are significantly expanded in CMV-seropositive elderly individuals.

We have also analysed T-bet and Eomes in CD8+ T-cells in relation to the expression of CD57. It has been described that CD57 is expressed in a significant percentage of CD8+ T-cells that increases with aging, in particular in CMV-seropositive donors, and has been considered a marker of dysfunctional senescent T-cells [38,39,40,41,42,43]. However, as indicated above for CD57+CD4+ T-cells, recent analysis of its expression in healthy young individuals supports the fact that CD57+CD8+ T-cells represent a subset of effector-memory and effector cells that are polyfunctional cells able to produce cytokines, including IFNγ [44].

It has also been shown that high levels of T-bet in CD8+ T-cells are associated with long-term resilience, low expression of inhibitory receptors, and protection from exhaustion in the experimental model of murine Lymphocytic Choriomeningitis Virus (LCMV) infection [19] and in elite non-progressor HIV-infected patients [20]. It has been proposed that in chronic viral infection, such as murine LCMV infection or human hepatitis C virus (HCV), CD8+ cells expressing T-bet^hi^ proliferate in response to persisting antigen, leading to Eomes^hi^ terminal differentiation [21] and supporting the relevance of these transcription factors in the maintenance of antiviral CD8+ T-cells during chronic viral infection. The analysis of T-bet and Eomes expression in CD8+ T-cells according to CMV-serostatus and age shows that the percentage of CD8+ T-cells positive for any of the transcription factors increases from CMV-seronegative young individuals to CMV-seropositive old donors. In particular, the percentage of CD8+ T-cells expressing T-bet but not Eomesis is higher in middle-aged and elderly CMV-seropositive donors compared with young CMV-seropositive individuals, whereas the percentage of those CD8+ T-cells coexpressing T-bet and Eomes is also higher in middle-aged CMV-seronegative compared with young CMV-seronegative donors. Higher percentages of CD8+ T-cells coexpressing T-bet and Eomes observed in old CMV-seropositive donors are found in both CD57−CD8+ and CD57+CD8+ T-cell subsets. No changes are observed in the percentage of Eomes single-positive CD8 cells (without T-bet) among the different groups considered. The majority of these cells do not express CD57 as the percentage of Eomes+CD57+CD8+ T-cells is negligible. These findings are in agreement with the recent observation showing that the majority (60–70%) of NKT-like effector CD8+ T-cells, defined by the expression of CD56, CD57 CD45RA, CD49d and KIR/NKG2A inhibitory receptors, coexpress Eomes and T-bet [45].

Two different subsets of CMV-specific CD8+ T-cells have been defined according to the expression of T-bet and Eomes [28]. Those expressing high levels of T-bet and lower levels of Eomes are the dominant populations and are highly efficient at the recognition of endogenously processed peptide–MHC complexes, although they show a low avidity for peptide–MHC, whereas those cells with lower levels of T-bet and high levels of Eomes represent the subdominant populations, are less efficient in the recognition of virus-infected cells, and have high peptide-MHC avidity [28]. Studies in mice have shown that the continuous stimulation of CMV-specific T-cells by persistent antigen exposure is required to maintain functional effector CD8+ T-cells responsible for protection against viral reactivation [46,47]. This sustained antigen exposure throughout life likely contributes to the age-associated oligoclonal expansion of two different subsets of CD8+ T-cells coexpressing graded levels of T-bet and of Eomes and reflects a balance between short-lived terminal CD57+ effector CD8+ T-cells and long-lived CD57− effector-memory CD8+ lymphocytes, characteristic of the periodic replenishment of specific T-cells necessary to achieve lifelong CMV immunity, as has been recently postulated for chronic infections [21,28].

The analysis of T-bet and Eomes in CD8 T-cells obtained from lung transplant recipients mismatched for CMV serostatus showed that patients showing higher ratios of T-bet:Eomes expression in CD8+ T-cells are able to control CMV replication versus with those with lower ratios that were viremic relapsers. CD8+ T-cells showing high ratios of T-bet:Eomes are better responders to CMV stimulation in terms of proliferation and, cytotoxicity and cytokine production, underscoring the importance of the T-bet:Eomes balance, with CMV-specific proliferation a key factor driving early T-bet expression and effector function in CD8+ T-cells during primary infection to establish immune control during early stages of CMV chronic infection [48].

In summary, our results support the relevance of CMV and aging in the expansion of highly differentiated CD4+ and CD8+ T-cells that differentially express T-bet and/or Eomes transcription factors. The expansion of these cells found in CMV-seropositive elderly donors suggest that the expression of these factors is essential not only for the development but also for the maintenance of an adequate pool of effector-memory and effector CD4+ and CD8+ T lymphocytes required to achieve lifelong immunity against chronic CMV infection. Further studies are required to define the impact of CMV and ageing on the expression of T-bet and Eomes in other T-cell subpopulations, such as the well-defined central memory, effector-memory or effector subsets, and the relevance of these transcription factors in the differentiation process of T-cells involved in the response to viruses and other pathogens.

## 4. Materials and Methods

### 4.1. Subjects

We studied 25 healthy donors stratified according to age and CMV serostatus (Table 1). However, all elderly donors included in the study were CMV-seropositive, as we were not able to recruit enough CMV-seronegative individuals given that the prevalence of CMV seropositivity in Spain in individuals over the age of 40 years is 80% [49] and, in our geographic area (Andalusia, Spain), about 99% of individuals over 65 years are CMV-seropositive.

All study participants provided informed written consent and met the following exclusion criteria: absence of diabetes, cancer, severe renal failure, severe liver disease, endocrine disorders, autoimmune diseases, or acute infectious disease; and they were not consuming drugs whose activity is known to modify the functions of the immune system. The ethical statement was approved by the Ethics Committee of the Reina Sofia University Hospital of Cordoba (Spain), on 25 February 2014 (reference number 2465). Peripheral blood from each subject was collected in lithium heparin tubes, followed by PBMCs isolation by density gradient centrifugation using Ficoll Histopage-1077 (Sigma-Aldrich, St. Louis, MO, USA). After isolation PBMCs were cryopreserved in FBS (Sigma-Aldrich) with 10% DMSO (Panreac Chemistry SAU, Barcelona, Spain) until experiments were performed.

### 4.2. CMV Serology

CMV-specific IgG and IgM was determined by automated enzyme-linked immunosorbent assay (ELISA) (DRG International, Mountainside, NY, USA) from plasma retrieved from all donors.

### 4.3. Flow Cytometry and Data Analysis

For cytometry experiments, cells were thawed in RPMI 1640 (Sigma-Aldrich) with 10% FBS (Gibco Life Technologies, Carlsbad, CA, USA) and subsequently placed in a 96-well plate at 2 × 10^6^ cells/mL concentration (250 µL final volume). Cells were then washed twice with PBS (4 °C) and surface stained for the following antibodies: anti-Live/Dead APC-Vio770 (LIVE/DEAD^®^ Fixable Near-IR Dead Cell Stain Kit, life technologies), anti-CD3 VioBlue (clone: BW264/56, MiltenyiBiotec, BergischGladbach, Germany), anti-CD57 Biotin-Anti-Biotin-Viogreen (clone: TB03, MiltenyiBiotec), anti-CD8 APC (clone: BW135/80, MiltenyiBiotec) and anti-CD4 PE-Vio770 (clone: M-T466, MiltenyiBiotec). Following cell fixation and permeabilisation using the Kit FoxP3 Staining Buffer Set (MiltenyiBiotec), intracellular staining of the transcription factors was performed according to the manufacturer’s instructions, with the following antibodies: anti-T-bet PerCP Cy5.5 (clone: 04-46, BD Pharmingen, San Diego, CA, USA) and anti-Eomes FITC (clone: WD1928, eBioscience, Waltham, MA, USA). All antibodies were titrated before use.

Samples were then acquired with a nine-parameter MACsQuant instrument (MiltenyiBiotech, BergischGladbach, Germany) and analysed with FlowJo v X 10.0.7 software (TreeStar, Portland, OR, USA). For data analysis, lymphocytes were gated according to their size and granularity (FSC vs. SSC), then forward scatter height (FSC-H) versus forward scatter area (FSC-A) to remove doublets. Within the singlets gate, Live T-cells (CD3+) were gated, followed by identification of the different T-cell subsets by confronting CD4 vs. CD8. Individual gates for T-bet+, Eomes+ and CD57+ T-cells were gated on each of these populations based on fluorescence minus one controls. A representative example of the gating strategy is shown in Figure 1. FlowJo’s Boolean combination gating options were used to analyse the coexpression of T-bet and Eomes and according to CD57 expression as well.

### 4.4. Statistical Analysis

Data were inspected for normal distribution using the Shapiro–Wilk test. No normality was found. The Mann–Whitney U nonparametric test was used to derive p-values for comparing data among the specific sample pairs. All statistical tests were performed with PASW Statistics v. 18. For graphs, GraphPad Prism (version 5.0, GraphPad Software, Inc., La Jolla, CA, USA) was used. Results are represented as scatter plots including the mean and SEM. The median and interquartile ranges of the coexpression analysis of the transcription factor T-bet and Eomes and CD57 on T-cell subsets is shown in the Supplementary Materials (Tables S1 and S2).

## Figures and Tables

**Table 1:** Demographics of studied individuals (*n* = 25).

Group	Age Group	Mean Age (SD)	Sex (Male/Female)
Young CMV-Seronegative	Young (18–35)	31 (6.82)	5/0
Young CMV-Seropositive	Young (18–35)	26 (2.92)	2/3
Middle-aged CMV-Seronegative	Middle-aged (40–60)	54.2 (8.79)	3/2
Middle-aged CMV-Seropositive	Middle-aged (40–60)	52.6 (5.32)	2/3
Elderly CMV-Seropositive	Elderly (>65)	74.6 (10.78)	4/1

**Table 2:** Percentage of CD4+ T-cells expressing T-bet and Eomes.

Transcription Factors	Group									
	Young CMV−		Young CMV+		Middle-Aged CMV−		Middle-Aged CMV+		Elderly CMV+	
	Mean	SEM	Mean	SEM	Mean	SEM	Mean	SEM	Mean	SEM
**EOMES+**	3.86	0.70	8.59	2.02	5.96	2.32	7.13	1.60	13.58	3.67
**T-bet+**	0.48	0.06	4.74	2.00	2.92	2.54	2.57	0.48	12.02	4.19

**Table 3:** Percentage of CD4^hi^CD8^lo^ T-cells expressing T-bet and Eomes.

Transcription Factors	Group									
	Young CMV−		Young CMV+		Middle-Aged CMV−		Middle-Aged CMV+		Elderly CMV+	
	Mean	SEM	Mean	SEM	Mean	SEM	Mean	SEM	Mean	SEM
**EOMES+**	39.01	8.05	58.05	6.98	43.22	6.24	41.53	8.35	63.87	11.93
**T-bet+**	7.35	1.66	33.81	14.48	17.93	12.17	26.85	7.00	51.87	13.01

**Table 4:** Percentage of CD8+ T-cells expressing T-bet and Eomes.

Transcription Factors	Group									
	Young CMV−		Young CMV+		Middle-Aged CMV−		Middle-Aged CMV+		Elderly CMV+	
	Mean	SEM	Mean	SEM	Mean	SEM	Mean	SEM	Mean	SEM
**EOMES+**	39.28	4.57	46.09	3.54	49.58	6.57	55.73	4.42	63.24	3.31
**T-bet+**	29.60	3.18	29.28	5.91	40.43	8.46	57.30	6.17	62.64	10.51

## Supplementary Materials

Supplementary materials can be found at www.mdpi.com/1422-0067/18/7/1391/s1.
