# Supplementary material for: Effect of Cytomegalovirus (CMV) and Ageing on T-Bet and Eomes Expression on T-Cell Subsets
Source: Int J Mol Sci. 2017 Jun 29;18(7):1391. doi: 10.3390/ijms18071391 (PMC5535884; doi:10.3390/ijms18071391)
Supplement: Supplementary file 1 [file ijms-18-01391-s001.pdf]

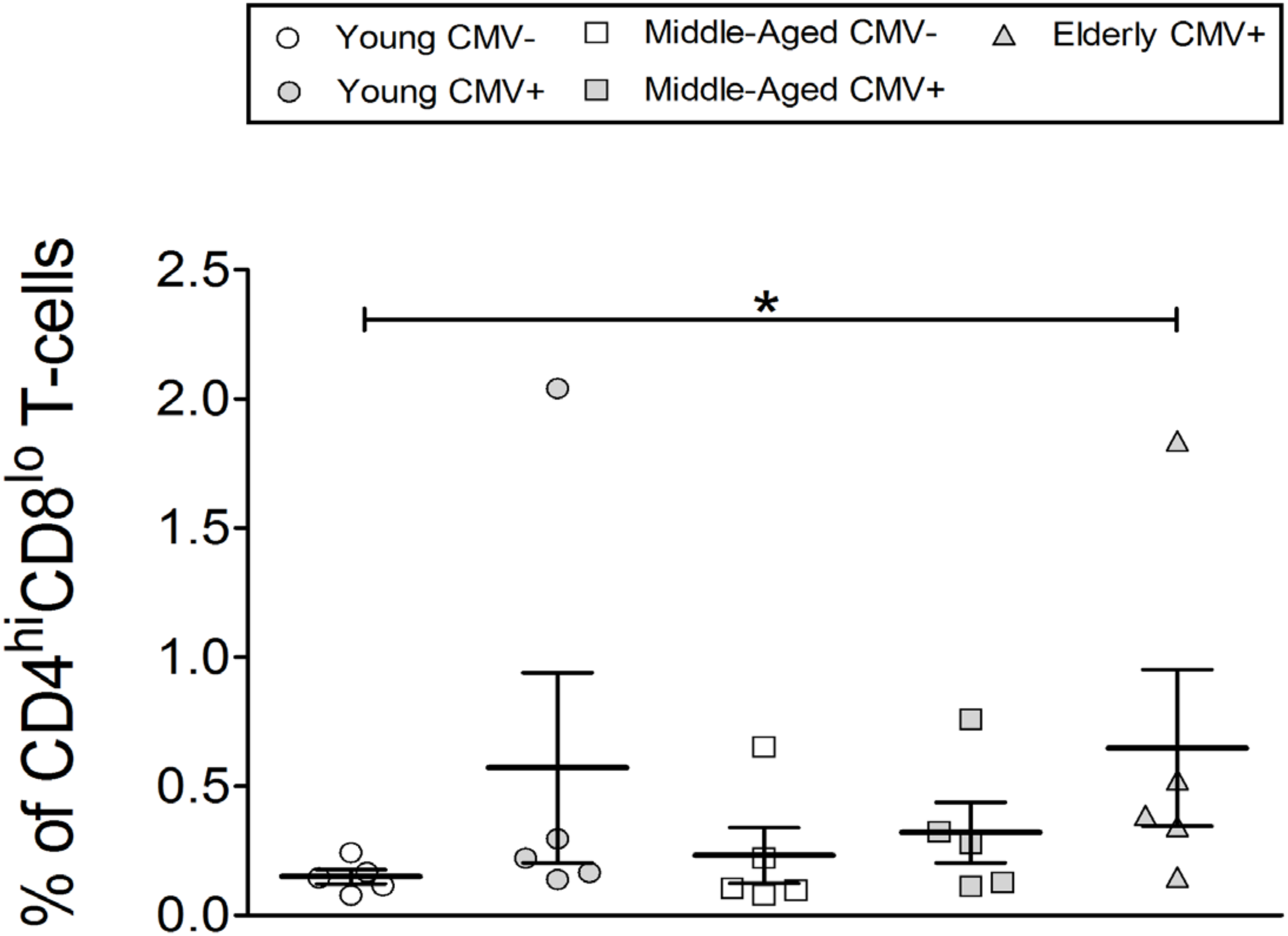

Figure S1. Percentage of $CD4^{hi}CD8^{lo}$ T-cells. Graph shows the percentage of $CD4^{hi}CD8^{lo}$ T-cells from healthy individuals (n=25), stratified by age and CMV serostatus. Scatter plot showing the mean and SEM. Results were considered significant at *$p < 0.05$, **$p < 0.01$, and ***$p < 0.001$.

Table S1. Median and InterQuartile Range (IQR) of the Co-expression analysis of T-bet and Eomes on T-cell subsets. A) CD4 and $CD4^{hi}CD8^{lo}$ phenotype								
**CMV status**	**CD4+ T-cells**	Median	Percentile 25	Percentile 75	**$CD4^{hi}CD8^{lo}$**	Median	Percentile 25	Percentile 75
**Young CMV-seronegative**	CD4+EOMES+	4,13	2,28	5,07	$CD4^{hi}CD8^{lo}$ EOMES+	40,74	34,04	42,59
	CD4+T-bet+	,51	,43	,55	$CD4^{hi}CD8^{lo}$ T-bet+	8,64	5,56	10,19
	CD4+EOMES+T-bet+	,18	,08	,28	$CD4^{hi}CD8^{lo}$ EOMES+T-bet+	5,56	3,70	8,33
	CD4+EOMES+T-bet-	3,83	2,19	4,79	$CD4^{hi}CD8^{lo}$ EOMES+T-bet-	37,04	23,40	37,04
	CD4+EOMES-T-bet+	,27	,20	,44	$CD4^{hi}CD8^{lo}$ EOMES-T-bet+	,00	,00	1,85
					$CD4^{hi}CD8^{lo}$ cells	,15	,12	,17
**Young CMV-seropositive**	CD4+EOMES+	8,51	6,26	12,04	$CD4^{hi}CD8^{lo}$ EOMES+	56,00	52,70	67,31
	CD4+T-bet+	4,49	,64	7,29	$CD4^{hi}CD8^{lo}$ T-bet+	24,00	7,69	51,23
	CD4+EOMES+T-bet+	3,59	,39	4,90	$CD4^{hi}CD8^{lo}$ EOMES+T-bet+	24,00	7,69	40,74
	CD4+EOMES+T-bet-	5,86	3,03	7,14	$CD4^{hi}CD8^{lo}$ EOMES+T-bet-	35,29	32,00	37,04
	CD4+EOMES-T-bet+	,90	,29	2,39	$CD4^{hi}CD8^{lo}$ EOMES-T-bet+	3,53	,00	10,49
					$CD4^{hi}CD8^{lo}$ cells	,22	,17	,30
**Middle-Aged CMV-seronegative**	CD4+EOMES+	3,46	3,29	7,43	$CD4^{hi}CD8^{lo}$ EOMES+	46,94	31,25	53,75
	CD4+T-bet+	,48	,26	,58	$CD4^{hi}CD8^{lo}$ T-bet+	8,16	2,50	10,77
	CD4+EOMES+T-bet+	,36	,10	,40	$CD4^{hi}CD8^{lo}$ EOMES+T-bet+	8,16	2,50	10,77
	CD4+EOMES+T-bet-	3,19	3,10	6,48	$CD4^{hi}CD8^{lo}$ EOMES+T-bet-	31,25	18,51	38,78
	CD4+EOMES-T-bet+	,16	,14	,22	$CD4^{hi}CD8^{lo}$ EOMES-T-bet+	,00	,00	2,08
					$CD4^{hi}CD8^{lo}$ cells	,11	,10	,22
**Middle-Aged CMV-seropositive**	CD4+EOMES+	6,69	4,70	9,81	$CD4^{hi}CD8^{lo}$ EOMES+	45,56	23,53	58,45
	CD4+T-bet+	2,48	2,45	3,03	$CD4^{hi}CD8^{lo}$ T-bet+	22,17	19,61	37,22
	CD4+EOMES+T-bet+	,81	,67	1,18	$CD4^{hi}CD8^{lo}$ EOMES+T-bet+	7,69	5,88	16,11
	CD4+EOMES+T-bet-	5,16	3,89	9,45	$CD4^{hi}CD8^{lo}$ EOMES+T-bet-	29,44	17,65	30,19
	CD4+EOMES-T-bet+	2,12	1,27	2,22	$CD4^{hi}CD8^{lo}$ EOMES-T-bet+	17,24	13,73	19,32
					$CD4^{hi}CD8^{lo}$ cells	,28	,13	,32
**Elderly CMV-seropositive**	CD4+EOMES+	13,37	11,97	13,51	$CD4^{hi}CD8^{lo}$ EOMES+	76,78	58,87	81,49
	CD4+T-bet+	9,01	8,33	18,01	$CD4^{hi}CD8^{lo}$ T-bet+	48,82	44,48	76,88
	CD4+EOMES+T-bet+	6,09	5,31	9,55	$CD4^{hi}CD8^{lo}$ EOMES+T-bet+	42,65	38,21	50,21
	CD4+EOMES+T-bet-	6,23	3,95	6,66	$CD4^{hi}CD8^{lo}$ EOMES+T-bet-	12,90	9,26	34,12
	CD4+EOMES-T-bet+	3,02	2,92	4,68	$CD4^{hi}CD8^{lo}$ EOMES-T-bet+	6,27	6,16	6,94
					$CD4^{hi}CD8^{lo}$ cells	,39	,34	,52

Table S1. Median and InterQuartile Range (IQR) of the Co-expression analysis of T-bet and Eomes on T-cell subsets. B) CD8 phenotype

**CMV status**	**CD8+ T-cells**	Median	Percentile 25	Percentile 75
**Young CMV-seronegative**	CD8+EOMES+	44,25	31,91	46,91
	CD8+T-bet+	31,98	30,69	33,94
	CD8+EOMES+T-bet+	24,78	14,58	25,56
	CD8+EOMES+T-bet-	19,10	18,69	22,13
	CD8+EOMES-T-bet+	8,67	4,77	9,16
**Young CMV-seropositive**	CD8+EOMES+	46,81	39,82	51,21
	CD8+T-bet+	21,53	20,22	39,17
	CD8+EOMES+T-bet+	17,75	15,10	32,68
	CD8+EOMES+T-bet-	23,06	14,54	24,72
	CD8+EOMES-T-bet+	5,12	4,36	6,12
**Middle-Aged CMV-seronegative**	CD8+EOMES+	50,28	38,45	53,20
	CD8+T-bet+	39,58	28,16	46,84
	CD8+EOMES+T-bet+	28,93	21,64	36,47
	CD8+EOMES+T-bet-	18,24	13,81	22,11
	CD8+EOMES-T-bet+	10,37	6,51	10,65
**Middle-Aged CMV-seropositive**	CD8+EOMES+	58,55	47,10	63,06
	CD8+T-bet+	66,64	44,82	66,85
	CD8+EOMES+T-bet+	42,50	32,55	49,42
	CD8+EOMES+T-bet-	13,64	12,13	14,55
	CD8+EOMES-T-bet+	12,28	12,27	17,42
**Elderly CMV-seropositive**	CD8+EOMES+	67,47	61,23	67,93
	CD8+T-bet+	68,87	67,27	72,45
	CD8+EOMES+T-bet+	49,98	45,03	54,48
	CD8+EOMES+T-bet-	16,20	13,45	18,49
	CD8+EOMES-T-bet+	18,89	17,97	22,24

Table S2. Median and InterQuartile Range (IQR) of the Co-expression analysis of T-bet, Eomes and CD57 on T-cell subsets A) CD4 and $CD4^{hi}CD8^{lo}$ phenotype

**CMV status**	**CD4+ T-cells**	Median	Percentile 25	Percentile 75	**$CD4^{hi}CD8^{lo}$**	Median	Percentile 25	Percentile 75
**Young CMV-seronegative**	CD4+Eomes+T-bet+CD57+	,02	,01	,03	$CD4^{hi}CD8^{lo}$ Eomes+T-bet+CD57+	,00	,00	,93
	CD4+Eomes+Tbet+CD57-	,17	,07	,17	$CD4^{hi}CD8^{lo}$ Eomes+Tbet+CD57-	5,56	2,47	7,41
	CD4+Eomes+Tbet-CD57+	,03	,02	,06	$CD4^{hi}CD8^{lo}$ Eomes+Tbet-CD57+	,93	,00	1,23
	CD4+Eomes+Tbet-CD57-	3,58	2,16	4,77	$CD4^{hi}CD8^{lo}$ Eomes+Tbet-CD57-	35,80	21,28	37,04
	CD4+Eomes-Tbet+CD57+	,01	,01	,02	$CD4^{hi}CD8^{lo}$ Eomes-Tbet+CD57+	,00	,00	1,23
	CD4+Eomes-Tbet+CD57-	,26	,19	,43	$CD4^{hi}CD8^{lo}$ Eomes-Tbet+CD57-	,00	,00	,00
	CD4+Eomes-Tbet-CD57+	,08	,05	,11	$CD4^{hi}CD8^{lo}$ Eomes-Tbet-CD57+	,00	,00	,00
**Young CMV-seropositive**	CD4+Eomes+T-bet+CD57+	,43	,14	2,57	$CD4^{hi}CD8^{lo}$ Eomes+T-bet+CD57+	1,92	,57	24,69
	CD4+Eomes+Tbet+CD57-	1,73	,25	2,33	$CD4^{hi}CD8^{lo}$ Eomes+Tbet+CD57-	12,05	5,77	16,05
	CD4+Eomes+Tbet-CD57+	,17	,08	,57	$CD4^{hi}CD8^{lo}$ Eomes+Tbet-CD57+	,57	,00	3,90
	CD4+Eomes+Tbet-CD57-	5,78	2,46	6,31	$CD4^{hi}CD8^{lo}$ Eomes+Tbet-CD57-	31,48	31,43	35,29
	CD4+Eomes-Tbet+CD57+	,09	,06	1,23	$CD4^{hi}CD8^{lo}$ Eomes-Tbet+CD57+	,00	,00	8,02
	CD4+Eomes-Tbet+CD57-	,81	,27	1,17	$CD4^{hi}CD8^{lo}$ Eomes-Tbet+CD57-	2,47	,00	3,53
	CD4+Eomes-Tbet-CD57+	,06	,05	,25	$CD4^{hi}CD8^{lo}$ Eomes-Tbet-CD57+	1,14	,62	1,18
**Middle-Aged CMV-seronegative**	CD4+Eomes+T-bet+CD57+	,05	,05	,06	$CD4^{hi}CD8^{lo}$ Eomes+T-bet+CD57+	1,54	,00	6,12
	CD4+Eomes+Tbet+CD57-	,30	,06	,35	$CD4^{hi}CD8^{lo}$ Eomes+Tbet+CD57-	2,50	2,04	9,23
	CD4+Eomes+Tbet-CD57+	,09	,03	,13	$CD4^{hi}CD8^{lo}$ Eomes+Tbet-CD57+	1,25	,00	2,04
	CD4+Eomes+Tbet-CD57-	3,07	3,06	5,87	$CD4^{hi}CD8^{lo}$ Eomes+Tbet-CD57-	31,25	15,80	36,73
	CD4+Eomes-Tbet+CD57+	,01	,01	,04	$CD4^{hi}CD8^{lo}$ Eomes-Tbet+CD57+	,00	,00	2,08
	CD4+Eomes-Tbet+CD57-	,15	,10	,21	$CD4^{hi}CD8^{lo}$ Eomes-Tbet+CD57-	,00	,00	,00
	CD4+Eomes-Tbet-CD57+	,03	,03	,06	$CD4^{hi}CD8^{lo}$ Eomes-Tbet-CD57+	,00	,00	2,08
**Middle-Aged CMV-seropositive**	CD4+Eomes+T-bet+CD57+	,37	,26	,39	$CD4^{hi}CD8^{lo}$ Eomes+T-bet+CD57+	,49	,00	2,22
	CD4+Eomes+Tbet+CD57-	,44	,40	,78	$CD4^{hi}CD8^{lo}$ Eomes+Tbet+CD57-	7,69	5,88	13,89
	CD4+Eomes+Tbet-CD57+	,20	,13	,20	$CD4^{hi}CD8^{lo}$ Eomes+Tbet-CD57+	2,56	1,97	2,78
	CD4+Eomes+Tbet-CD57-	4,96	3,69	9,42	$CD4^{hi}CD8^{lo}$ Eomes+Tbet-CD57-	25,12	15,69	26,67
	CD4+Eomes-Tbet+CD57+	,60	,10	,63	$CD4^{hi}CD8^{lo}$ Eomes-Tbet+CD57+	3,94	3,33	8,70
	CD4+Eomes-Tbet+CD57-	1,32	,67	1,60	$CD4^{hi}CD8^{lo}$ Eomes-Tbet+CD57-	10,63	3,92	13,30
	CD4+Eomes-Tbet-CD57+	,27	,09	,30	$CD4^{hi}CD8^{lo}$ Eomes-Tbet-CD57+	3,45	3,33	3,92
**Elderly CMV-seropositive**	CD4+Eomes+T-bet+CD57+	3,22	3,22	6,47	$CD4^{hi}CD8^{lo}$ Eomes+T-bet+CD57+	9,25	6,64	40,43
	CD4+Eomes+Tbet+CD57-	2,87	2,10	3,09	$CD4^{hi}CD8^{lo}$ Eomes+Tbet+CD57-	21,30	9,79	28,96
	CD4+Eomes+Tbet-CD57+	,78	,72	1,10	$CD4^{hi}CD8^{lo}$ Eomes+Tbet-CD57+	3,32	2,78	3,58
	CD4+Eomes+Tbet-CD57-	4,97	3,24	5,56	$CD4^{hi}CD8^{lo}$ Eomes+Tbet-CD57-	12,90	6,48	30,81
	CD4+Eomes-Tbet+CD57+	1,60	1,19	1,87	$CD4^{hi}CD8^{lo}$ Eomes-Tbet+CD57+	3,70	2,99	4,27
	CD4+Eomes-Tbet+CD57-	1,72	1,42	1,98	$CD4^{hi}CD8^{lo}$ Eomes-Tbet+CD57-	3,24	2,15	3,28
	CD4+Eomes-Tbet-CD57+	,50	,42	,56	$CD4^{hi}CD8^{lo}$ Eomes-Tbet-CD57+	,90	,00	1,90

Table S2. Median and InterQuartile Range (IQR) of the Co-expression analysis of T-bet, Eomes and CD57 on T-cell subsets. B) CD8 phenotype

**CMV status**	**CD8+ T-cells**	Median	Percentile 25	Percentile 75
**Young CMV-seronegative**	CD8+Eomes+T-bet+CD57+	7,87	7,20	12,03
	CD8+Eomes+Tbet+CD57-	11,98	11,81	12,76
	CD8+Eomes+Tbet-CD57+	,96	,51	1,21
	CD8+Eomes+Tbet-CD57-	18,81	18,18	20,86
	CD8+Eomes-Tbet+CD57+	3,09	1,74	5,70
	CD8+Eomes-Tbet+CD57-	3,92	3,46	5,59
	CD8+Eomes-Tbet-CD57+	2,56	2,04	3,57
**Young CMV-seropositive**	CD8+Eomes-Tbet-CD57-	4,31	3,30	8,79
	CD8+Eomes+T-bet+CD57+	14,45	11,81	23,89
	CD8+Eomes+Tbet+CD57-	,45	,42	,50
	CD8+Eomes+Tbet-CD57+	22,77	14,04	24,27
	CD8+Eomes+Tbet-CD57-	2,35	1,70	2,43
	CD8+Eomes-Tbet+CD57+	2,97	2,29	3,68
	CD8+Eomes-Tbet+CD57-	4,55	2,27	5,26
**Middle-Aged CMV-seronegative**	CD8+Eomes-Tbet-CD57+	3,33	3,31	11,52
	CD8+Eomes-Tbet-CD57-	19,07	17,41	19,38
	CD8+Eomes+T-bet+CD57+	,56	,22	,90
	CD8+Eomes+Tbet+CD57-	15,96	13,59	17,34
	CD8+Eomes+Tbet-CD57+	2,36	,60	5,92
	CD8+Eomes+Tbet-CD57-	4,73	3,23	6,09
	CD8+Eomes-Tbet+CD57+	10,00	4,35	25,00
**Middle-Aged CMV-seropositive**	CD8+Eomes-Tbet+CD57-	17,31	16,77	24,07
	CD8+Eomes-Tbet-CD57+	22,61	20,16	25,18
	CD8+Eomes-Tbet-CD57-	,83	,50	1,25
	CD8+Eomes+T-bet+CD57+	12,39	11,37	12,92
	CD8+Eomes+Tbet+CD57-	8,98	8,19	10,32
	CD8+Eomes+Tbet-CD57+	4,39	4,09	7,10
	CD8+Eomes+Tbet-CD57-	,79	,69	1,41
**Elderly CMV-seropositive**	CD8+Eomes-Tbet+CD57+	21,27	16,68	24,39
	CD8+Eomes-Tbet+CD57-	28,35	22,07	28,71
	CD8+Eomes-Tbet-CD57+	1,78	1,14	1,82
	CD8+Eomes-Tbet-CD57-	14,37	12,54	16,67
	CD8+Eomes+T-bet+CD57+	12,28	11,89	12,36
	CD8+Eomes+Tbet+CD57-	5,61	3,24	7,01
	CD8+Eomes+Tbet-CD57+	,58	,44	,98
